# Utilising Electronic PROMs to Measure a Change in Health Following Elective Laparoscopic Cholecystectomy: A Feasibility Study

**DOI:** 10.1007/s00268-022-06588-9

**Published:** 2022-05-24

**Authors:** Prita Daliya, Dileep N. Lobo, Simon L. Parsons

**Affiliations:** 1 Trent Oesophago-Gastric Unit, Nottingham University Hospitals NHS Trust, , City Hospital Campus, Hucknall Road, Nottingham, UK; 2 Gastrointestinal Surgery, Nottingham Digestive Diseases Centre and National Institute for Health Research (NIHR) Nottingham Biomedical Research Centre, Nottingham University Hospitals NHS Trust and University of Nottingham, Queen’s Medical Centre, Nottingham, UK; 3 MRC Versus Arthritis Centre for Musculoskeletal Ageing Research, School of Life Sciences, University of Nottingham, Queen’s Medical Centre, Nottingham, UK

## Abstract

**Background:**

The collection of patient-reported outcome measures (PROMs) has many benefits for clinical practice. However, there are many barriers that prevent them from becoming a part of routine clinical care. The aim of this feasibility study was to pilot the use of a digital platform to facilitate the routine collection of pre- and post-operative electronic PROMs (ePROMs) in participants undergoing elective laparoscopic cholecystectomy and to validate the use of existing patient-reported outcomes for our population.

**Methods:**

Participants scheduled for elective laparoscopic cholecystectomy were asked to complete digital versions of the Otago gallstones Condition-Specific Questionnaire (CSQ), and the RAND 36-item health survey (SF36). An assessment of methodological quality of ePROM questionnaires was also performed.

**Results:**

Preoperative ePROMs were completed by 200 participants undergoing laparoscopic cholecystectomy. Post-operatively attrition was high (completion at 30 days, 3 months, and 6months: *n* = 61, 54, and 38, respectively) due to difficulties accessing our ePROMs portal. Of those able to complete, a significant improvement in quality of life was seen across all health domains post-operatively when compared with baseline preoperative values for both disease-specific and generic PROMs. Methodological quality was assessed as good to excellent in both digital questionnaires.

**Conclusion:**

The collection of ePROMs is possible with current technological advances. Although it may be an acceptable, and convenient process for patients, and a useful measure of quality-of-life trends for clinicians, further developmental work is necessary to improve accessibility for patients, improve compliance, and reduce reporting bias from high attrition rates.

**Supplementary Information:**

The online version contains supplementary material available at 10.1007/s00268-022-06588-9.

## Introduction

Gallstones are a common problem, affecting 10–15% of adults over the age of 18 years, with around 60,000 cholecystectomies being performed in England alone [1]. Symptomatic gallstone disease can pose a significant health and societal burden, impacting physical well-being, and an individual’s ability to work [2]. Despite the clear benefits of surgery and the potential economic savings resulting from preventing further morbidity from gallstones, laparoscopic cholecystectomy is not without risks, some of these being associated with a significant reduction in quality of life [3, 4].

Patient-reported outcomes (PROs) are used as a subjective measure for a patient’s health-related quality of life (HRQoL), at a set point in time, and from the patient’s perspective [5]. The use of validated questionnaires or patient-reported outcome measures (PROMs) has been mandatory practice in the UK National Health Service’s (NHS) national PROMs programme since 2009 [6, 7]. HRQoL data collected for patients undergoing hip and knee replacement surgery, groin hernia repair, and varicose vein surgery have been used to enable providers, commissioners, and stakeholders to make informed changes to service delivery which can then be used to improve clinical outcomes following intervention or surgery [6, 8]. The collection of these data often involves postal surveys which are time-consuming and costly [9, 10]. However, recent studies, including a meta-analysis, have demonstrated no major differences or limitations in using electronically collected PROMs (ePROMs) compared with more traditional paper PROMs [10–13].

Given current deficiencies in the NHS budget, and a recent government drive to a paperless NHS, ePROMs offer a functional solution for the routine use of PROMs and incorporation into Digital Health Records [14, 15].

The aim of this feasibility study was to pilot the use of a digital platform to facilitate the routine collection of pre- and post-operative ePROMs in participants undergoing elective laparoscopic cholecystectomy to calculate a change in HRQoL. Secondary objectives were to validate the use of existing PROs for our population by appraising their methodological quality.

## Methods

### PRO selection

Several PROs relevant to patients undergoing laparoscopic cholecystectomy have previously been identified and appraised [16–18]. However, due to the commercial nature of many trademarked PROs and the cost of licensing, we were limited in the selection available for this study. We took guidance from existing literature on the reporting of HRQoL studies and selected both disease-specific and generic PROs and planned for PRO validation within the study [19, 20].

Due to the inclusion of two PROs, and to prevent question fatigue, the 6-item Otago gallstones condition-specific questionnaire (CSQ) [21] was identified as the disease-specific PRO, and the RAND 36-Item health survey - version 1.0 (SF36) [22], as the generic PRO. Each of the items in both the CSQ and SF36 has one of 5 potential answers based on a Likert scale. Each answer has an allocated score: for CSQ 0 = best health, and 100 = worst health [21], and for SF36 0 = worst health, and 100 = best health [22]. Further domain scores can also be calculated for each PRO by combining and averaging specific items.

### Variables

Participant and hospital variables collected included details on age, sex, Charlson comorbidity index [23], body mass index (BMI), and total length of hospital stay. Data were also collected on 30-day outcomes, specifically post-operative complications, re-attendances and readmission to hospital, a return to theatre, and mortality.

For comparison with our local population, data were also collected on all other patients undergoing laparoscopic cholecystectomy in the study time who opted not to participate in our PROMs study.

### Outcomes

The main outcome measures included generic and disease-specific quality of life. PROs were offered to participants preoperatively, and post-operatively at 30 days, 3 months, and 6 months. Domain scores were calculated for each completed PRO, with post-operative results compared directly with baseline (preoperative) results.

The validity of PROs as ePROs was assessed by appraising their methodological quality within the study [16, 24]. Response rates, reliability, internal consistency, floor and ceiling effects, and responsiveness were all assessed within the study sample. Cross-correlation between CSQ and SF36 was also performed.

### Statistical analysis

Data were analysed using GraphPad Prism® version 8.3.0. (GraphPad Software LLC, San Diego, CA, USA). Nonparametric tests were used to compare differences between groups as results were not normally distributed. Fisher’s exact or Chi-square tests were used for categorical variables and the Mann–Whitney U test for continuous variables. Spearman’s r was taken as a measure of correlation and reliability between scales, and Cronbach’s alpha for internal consistency assessment. Differences were considered significant at *p* < 0.05.

### Ethics and reporting

Study participants were recruited to participate in this study on referral to hospital for management of symptomatic gallstones. On receiving study information either postally or in person, each participant indicated their consent to participate digitally. The study was performed as part of a larger PhD project sponsored by Nottingham University Hospitals NHS Trust, through a collaboration with EIDO Healthcare Limited and the Royal College of Surgeons of England. The study proposal was appraised by the confidentiality advisory group: 16/CAG/0045, with public and patient involvement, and research ethics committee approval: 16/SW/0088. It was registered with ClinicalTrials.gov: NCT02810860. The study was conducted and reported in accordance with the Strengthening the Reporting of Observational Studies in Epidemiology (STROBE) guidelines for observational studies [25].

## Results

### Participant recruitment and study demographics

**Fig. 1:**
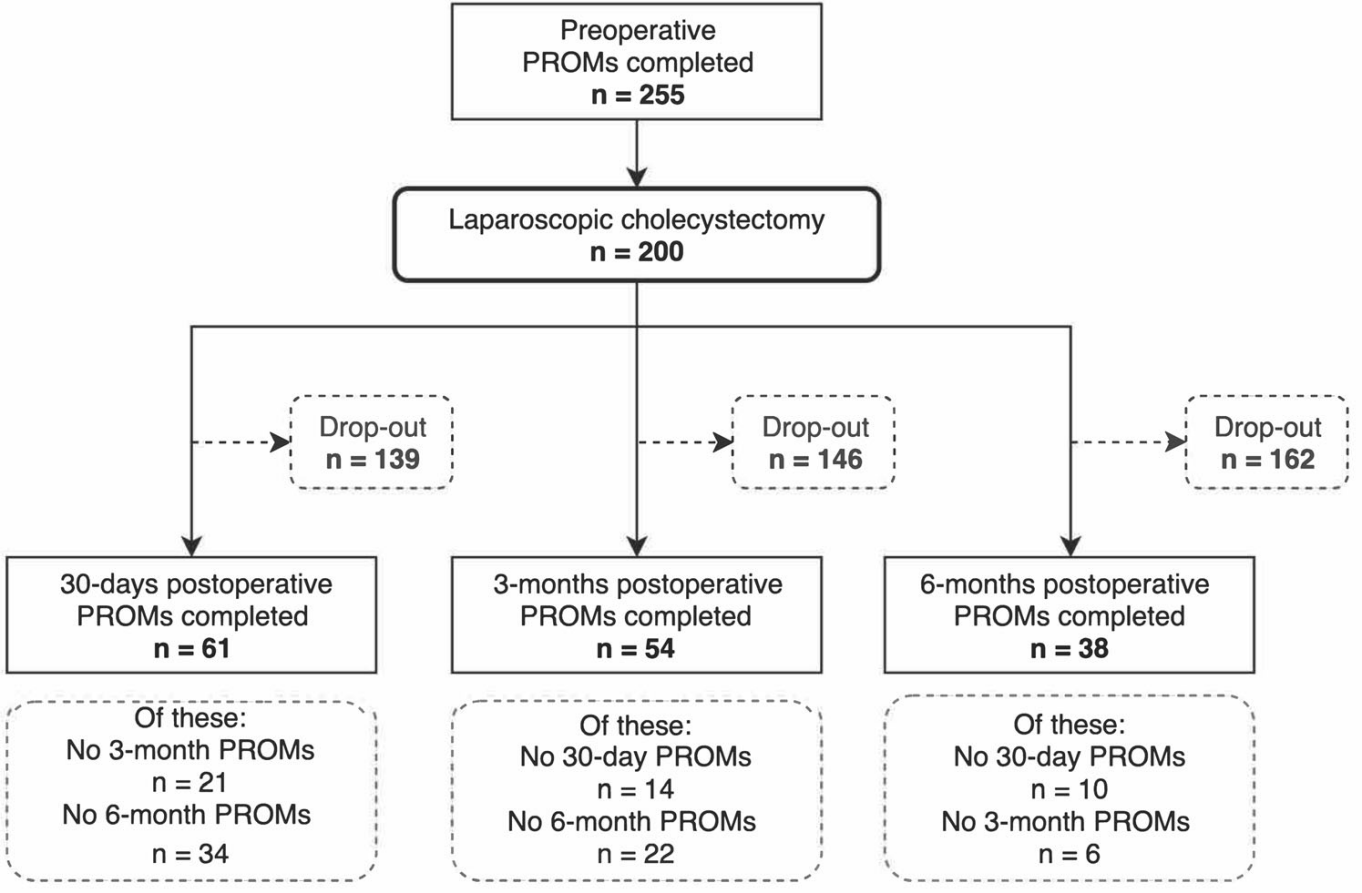
Participant recruitment—STROBE flow chart

A total of 255 eligible patients who were invited to participate in the study and use our digital interface completed the preoperative PROMs surveys between 6 September 2016 and 16 April 2018. Only 200 of these went on to undergo a laparoscopic cholecystectomy. These participants completed their preoperative survey a median of 86.5 days (IQR: 51–141.3) before laparoscopic cholecystectomy. The remaining participants were managed non-operatively because of patient choice, incorrect initial diagnosis, or being medically unfit for surgery. Only a small proportion of the 200 participants who completed their preoperative PROMs questionnaire completed the post-operative surveys at 30 days, 3 months, and 6 months (30.5, 27.0, 19.0%, respectively) (Fig. 1).

None of the 255 participants who completed the preoperative survey asked to withdraw from the study. Most participants, who opted not to complete a post-operative survey, maintained contact with the research team via a support email and described the complexity of accessing our ePROMs interface as the main reason for non-participation.

Participant demographics are described in Table 1. Participants in the study group were statistically younger than our local population. There were no other significant differences between the two groups.

**Table 1:** Participant demographics

		Study sample *n* = 200	Local population *n* = 341	*p* value
Age (years)	Mean (SD) years	46.2 (14.3)	49.4 (15.9)	0.02*
	IQR	34.6 – 56.8	35.4 – 62.0	
Sex	Female	163 (81.5)	292 (85.6)	0.22†
	Male	37 (18.5)	49 (14.4)	
Charlson comorbidity index score	0	157 (78.5)	248 (72.7)	0.05‡
	1	22 (11.0)	44 (12.9)	
	2	13 (6.5)	32 (9.4)	
	≥ 3	8 (4.0)	17 (5.0)	
Body mass index (kg/m^2^)	Median	29.5	29.7	0.64*
	IQR	25.5 – 35.9	26.0 – 34.3	
Length of stay	0 days	123 (61.5)	212 (62.2)	0.18^‡^
	1 day	63 (31.5)	92 (27.0)	
	2 days	6 (3.0)	8 (2.3)	
	≥3 days	8 (4.0)	29 (8.5)	

Values expressed as number (%) unless otherwise stated. *SD* Standard deviation. *IQR* Inter-quartile range. *Mann–Whitney U test, †Fisher’s exact test, ‡Chi-square test

### 30-Day outcomes

Thirty-day outcomes were collected for all participants (Table 2). Most attendances for complications [61.5% (16 of 26) 30-day complications] were managed in an ambulatory setting and did not require overnight admission. Admission for treatment other than surgery or radiological drainage included intravenous hydration or medications (antibiotics, anti-emetics, analgesia), wound management, or a period of observation.

**Table 2:** 30-day post-operative outcomes

	Study sample *n* = 200	Local population *n* = 341	*p* value
Complications	26 (13.0)	42 (12.3)	0.89†
Intra-abdominal collection	1 (0.5)	5 (1.5)	
Wound infection	9 (4.5)	17 (5.0)	
Other wound problem^a^	3 (1.5)	4 (1.2)	
Post-operative bleed	1 (0.5)	0	
Suspected chyle leak	1 (0.5)	0	
Suspected bile leak	1 (0.5)	2 (0.6)	
Small bowel injury	1 (0.5)	0	
Haemorrhagic pancreatitis	1 (0.5)	0	
Persistent pain	5 (2.5)	10 (2.9)	
Other^b^	3 (1.5)	4 (1.2)	
Return to theatre	4 (2.0)	2 (0.6)	0.20†
Laparoscopic washout and drain	4 (2.0)	2 (0.6)	
Laparotomy	2 (1.0)	2 (1.0)	
Re-admissions	7 (3.5)	13 (3.8)	1.00†
Return to theatre	1 (0.5)	2 (0.6)	
Percutaneous drain	1 (0.5)	3 (0.9)	
Intravenous treatment	4 (2.0)	7 (2.0)	
Observation	1 (0.5)	1 (0.3)	
Re-admission length of stay			
Mean days (SD)	1.6 (2.8)	1.7 (3.2)	0.94‡
Median days (IQR)	0 (0.0–2.2)	0 (0.0–3.0)	
Mortality	0	0	–

^a^Wound dehiscence, wound haematoma, stitch sinus

^b^Post-operative nausea and vomiting, diarrhoea, allergic reaction, hernia, pancreatitis, urine infection

†Fisher’s exact test, ‡ Mann–Whitney U test

### Patient-reported outcome measures

Quality-of-life domain scores were calculated from item scores for both CSQ (Table 3) and SF36 (Table 4). Compared with baseline preoperative values, statistically significant improvements in quality of life were seen in most domains in both CSQ and SF36.

**Table 3:** Disease-specific quality-of-life scores (CSQ)

HRQoL domains	Preoperative *n* = 200	30 days *n*=61	3 months *n* = 54	6 months *n* = 38
Physical functioning	55.4 (21.6)	17.5 (19.8)*	18.6 (19.8)*	16.7 (20.6)*
Systemic functioning	61.1 (32.6)	31.6 (31.9)*	18.1 (25.4)*	18.4 (30.0)*
Emotional functioning	48.9 (32.3)	15.6 (25.9)*	16.7 (28.7) *	7.9 (20.2)*
Social functioning	37.7 (27.9)	21.8 (22.4)*	7.9 (14.8)*	7.9 (12.7)*
Overall	75.4 (23.3)	19.3 (26.0)*	16.2 (22.9)*	16.4 (29.2)*

Disease-specific PROMs measure utilising the Otago gallstones condition-specific questionnaire (CSQ). PRO scores are represented as mean values (standard deviation), where 0 = best health, 100 = worst health

*p<0.05 using Wilcoxon test of matched pairs, comparing the baseline (preoperative) value with each respective post-operative time point in turn (i.e. only 61 results analysed at 30 days, 54 at 3 months, and 38 at 6 months)

**Table 4:** Generic quality-of-life scores (SF36)

HRQoL domains	Preoperative *n* = 200	30 days *n*=61	3 months *n* = 54	6 months *n* = 38
Physical functioning	68.6 (28.3)	75.5 (29.6)	81.4 (28.9)*	85.9 (19.3)*
Role limitations due to physical health	35.0 (41.4)	41.4 (43.0)	69.4 (40.8)*	78.3 (36.4)*
Role limitations due to emotional problems	53.7 (43.0)	67.8 (42.1)	80.2 (35.2)*	71.9 (39.9)
Energy/fatigue	36.5 (22.8)	51.9 (26.1)*	56.7 (25.4) *	56.8 (25.8)*
Emotional well-being	60.1 (21.5)	73.7 (22.5)*	74.3 (21.9)*	73.5 (21.7)
Social functioning	57.1 (28.9)	73.2 (26.0)*	78.5 (27.6)*	83.9 (24.1)*
Pain	38.2 (26.1)	57.5 (26.0)*	70.6 (29.5)*	76.9 (27.1)*
General health	52.5 (21.1)	64.7 (25.1)*	60.6 (26.0)	63.0 (22.6)
Change in health	36.4 (24.4)	65.2 (28.6)*	62.5 (29.4)*	69.1 (29.3)*

Generic PROMs measure utilising the RAND 36-Item health survey 1.0. Values expressed as a number (standard deviation), where 0 = worst health, 100 = best health

*p<0.05 using Wilcoxon test of matched pairs, comparing the baseline (preoperative) value with each respective post-operative time point in turn (i.e. only 61 results analysed at 30 days, 54 at 3 months, and 38 at 6 months)

**Fig. 2:**
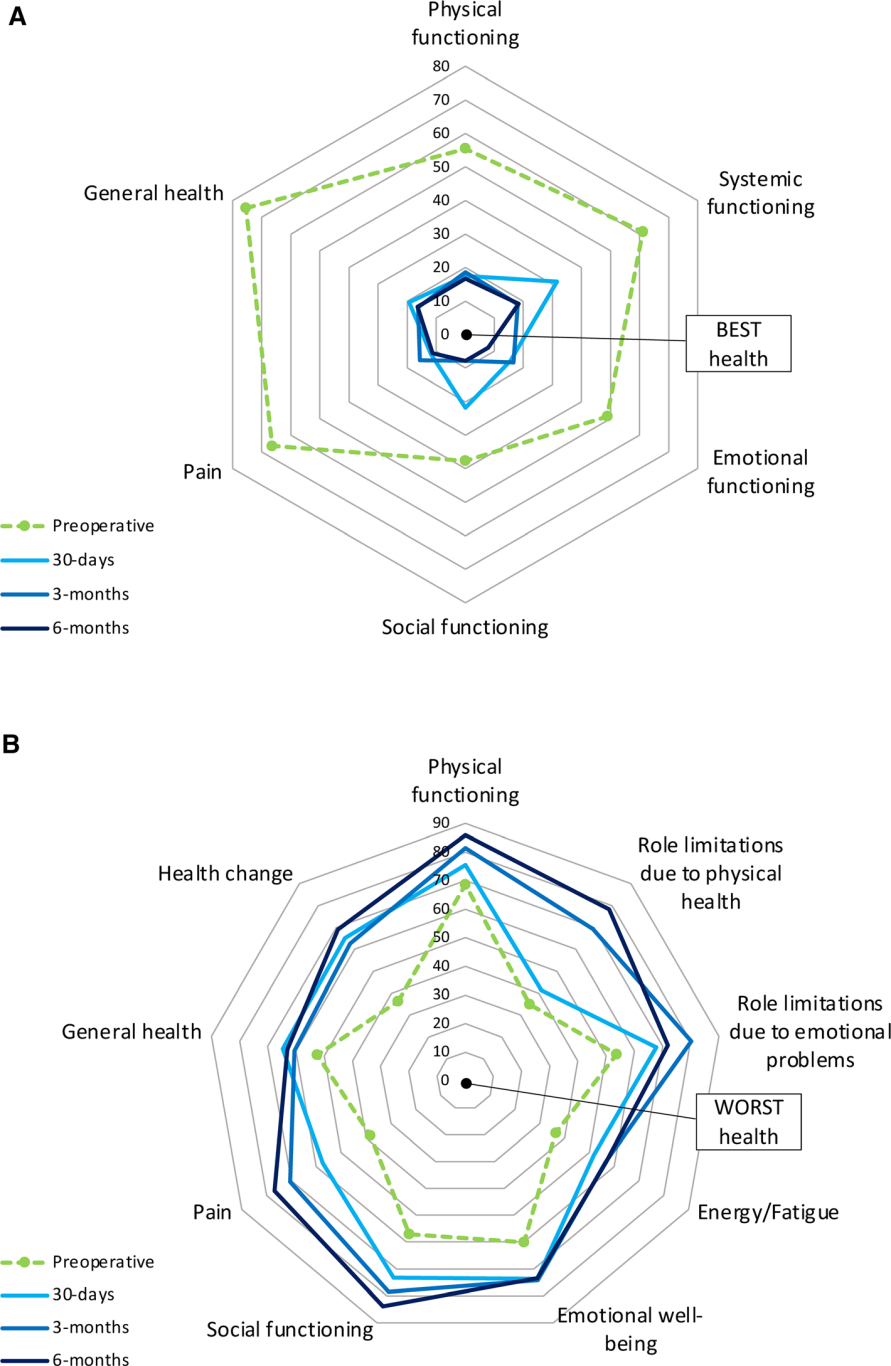
**a** Change in health measured by CSQ. Visual representation of change in disease-specific HRQoL between each time point. Preoperative scores are represented in green, and post-operative scores are represented in blue. The central point of the radar chart represents best possible health **b** Change in health measured by RAND SF36. Visual representation of change in generic HRQoL between each time point. Preoperative scores are represented in green, and post-operative scores are represented in blue. The outer most edge of the radar chart represents best possible health.

The change in HRQoL before and after surgery is mapped in Fig. 2. These radar plots demonstrate that all participants experienced an improvement in HRQoL, in all domains, between preoperative and post-operative time points as demonstrated by no crossover between blue (solid) and green (dashed) plot lines.

### Completion rates

The digital nature of our ePROMs platform meant that surveys were either completed in their entirety or not all and there were no missing items within individual returned surveys.

### Reliability

Test–retest correlations were performed between post-operative groups using Spearman’s coefficient of correlation on PROMs taken at 30 days and 3 months, and at 3 and 6 months post-operatively, and showed moderate-to-strong reliability (Table 5).

**Table 5:** Assessment of reliability

	30 days versus 3 months			3 months versus 6 months		
	Spearman’s r	95% CI	*p* value	Spearman’s r	95% CI	*p* value
CSQ						
Physical functioning	0.35	0.03–0.60	0.03	0.52	0.20–0.74	<0.01
Systemic functioning	0.53	0.25–0.72	<0.01	0.59	0.29–0.78	<0.01
Emotional functioning	0.65	0.42–0.80	<0.01	0.54	0.23–0.75	<0.01
Social functioning	0.41	0.10–0.65	<0.01	0.65	0.38–0.82	<0.01
Overall	0.33	0.01–0.59	0.04	0.86	0.72–0.93	<0.01
SF36						
Physical functioning	0.62	0.37–0.78	<0.01	0.70	0.45–0.80	<0.01
Role limitations due to physical health	0.47	0.18–0.69	<0.01	0.83	0.67–0.92	<0.01
Role limitations due to emotional problems	0.47	0.18–0.69	<0.01	0.47	0.13–0.71	<0.01
Energy/fatigue	0.77	0.60–0.88	<0.01	0.82	0.66–0.91	<0.01
Emotional well-being	0.75	0.56–0.86	<0.01	0.75	0.54–0.87	< 0.01
Social functioning	0.80	0.65–0.89	<0.01	0.53	0.21–0.74	<0.01
Pain	0.66	0.44–0.81	<0.01	0.79	0.60–0.89	<0.01
General health	0.87	0.76–0.93	<0.01	0.70	0.46–0.85	<0.01
Change in health	0.58	0.32–0.76	<0.01	0.56	0.26–0.77	<0.01

Spearman’s *r*: < 0.30 = weak, 0.30–0.49 = moderate, ≥ 0.5 = strong reliability. *CI* Confidence Interval

### Internal consistency

Homogeneity within PROs or internal consistency was good or excellent (Table 6).

**Table 6:** Assessment of internal consistency

	Number of items	Sum of variances	Variance of total scores	Cronbach’s alpha
*CSQ*				
Physical functioning	6	5499.19	16,793.75	0.81
Social functioning	3	3059.44	6975.19	0.84
SF36				
Physical functioning	10	12,055.31	79,873.44	0.94
Role limitations due to physical health	4	9037.50	27,300.00	0.89
Role limitations due to emotional problems	3	7288.50	16,579.00	0.84
Energy/fatigue	4	2725.11	8292.79	0.90
Emotional well-being	5	3467.29	11,537.75	0.87
Social functioning	2	1883.67	3316.11	0.86
Pain	2	1549.75	2716.75	0.86
General health	5	3989.48	11,131.23	0.80

Cronbach’s alpha: > 0.7 = acceptable, > 0.8 = good, > 0.9 = excellent internal consistency

### Floor and ceiling effects

To measure the ability of the PROs to identify population variation, floor and ceiling effects were assessed in both HRQoL instruments. Whilst floor and ceiling effects were seen in both CSQ and SF36 domain measurements, there were few instances exceeding > 15% (Table 7).

**Table 7:** Assessment of floor and ceiling effects

	Floor effect %	Ceiling effect %
CSQ		
Physical functioning	0	2.0
Systemic functioning	23.5	12.0
Emotional functioning	11.0	17.5
Social functioning	3.0	12.5
Overall	34.0	1.0
SF36		
Physical functioning	1.5	19.5
Role limitations due to physical health	50.0	22.5
Role limitations due to emotional problems	30.5	40.5
Energy/fatigue	4.0	0.5
Emotional well-being	0.5	0.5
Social functioning	2.5	15.0
Pain	11.0	4.5
General health	0	0
Change in health	15.0	4.0

Floor/ceiling effects: > 15% = poor, < 15% = adequate, 0 = excellent

### Responsiveness and effect sizes

The ability to capture statistically significant changes in HRQoL over time was assessed by calculating effect sizes (Table 8). Effect sizes were classified as < 0.3, 0.5, or > 0.8, as small, medium, or large. Scores for CSQ were converted to a positive scale much like SF36 for the purposes of analysis.

**Table 8:** Assessment of responsiveness

HRQoL domains	30 days *n*=61	3 months *n*=54	6 months *n*=38
CSQ			
Physical functioning	1.8	1.7	1.8
Systemic functioning	0.9	1.3	1.3
Emotional functioning	1.0	1.0	1.3
Social functioning	0.6	1.1	1.1
Overall	2.4	2.5	2.5
SF36			
Physical functioning	0.2	0.4	0.6
Role limitations due to physical health	0.1	0.8	1.0
Role limitations due to emotional problems	0.3	0.6	0.4
Energy/fatigue	0.7	0.9	0.9
Emotional well-being	0.6	0.7	0.6
Social functioning	0.6	0.7	0.9
Pain	0.7	1.2	1.5
General health	0.6	0.4	0.5
Change in health	1.2	1.0	1.3

Responsiveness:<0.3=small, ~ 0.5=medium, ≥0.8=large

### Cross-correlation between CSQ and SF36

Assessment of cross-correlation between the disease-specific and generic PROs showed moderate-to-strong reliability across the domains of ‘Physical Functioning’, ‘Systemic Functioning’, ‘Emotional Functioning’, ‘Social Functioning’, and ‘Overall health’ (Supplementary Figs. 1–5 and Supplementary Table 1).

## Discussion

This novel feasibility study demonstrates the potential benefits of using a digital platform to collect real-time quality-of-life information utilising ePROMs. Despite initial problems in implementing our digital platform, and difficulties with user access, the process was deemed acceptable by most participants. However, these difficulties did lead to a significant attrition rate in the completion of post-operative questionnaires and resultant reporting bias.

### Participant recruitment

Participants described the remote process of initial contact and invitation to the study positively and were pleased to have a study helpline for the digital platform. Whilst a significant number of participants subsequently dropped out of the study, discussion with those who contacted the helpline described this as mainly due to difficulties with Website onboarding due to the multiple security measures which were requested by our local information governance team (email, secure password, two type-sensitive memorable words). Of the 80 participants who contacted the user helpline, 64 described difficulties with either site registration or log-in (requiring support on between 1 and 6 occasions).

Participants subsequently recruited and retained in the study were found to be younger and have fewer comorbidities than those from a similar cohort that underwent laparoscopic cholecystectomy during the same time frame but out-with the study. This generational difference in our study group was in keeping with other studies utilising ePROMs [13].

All other study demographics demonstrated no significant differences between the two groups, indicating that the study group was representative of our local population.

### Disease-specific PROMs

Statistically significant improvements in disease-specific HRQoL were seen across all survey items, and all quality-of-life domains following laparoscopic cholecystectomy when compared with baseline preoperative values. Marked changes with over twofold improvements in quality-of-life scores were evident at 30 days post-operatively in all domains.

Continued improvement was seen in emotional functioning and social functioning at 6 months post-operatively, whereas physical functioning, systemic functioning, and overall health either peaked or plateaued at 3 months post-operatively. Post-operative HRQoL did not fall to preoperative levels in any of the domains demonstrating that the continued benefit laparoscopic cholecystectomy had on participants.

### Generic PROMs

Post-operative improvements were seen in all domains for generic HRQoL measures after laparoscopic cholecystectomy. This finding was in keeping with other non-UK-based studies on HRQoL [26, 27]. Continued significant improvement in quality of life was seen at 30 days, 3 months, and 6 months for fatigue, social functioning, and pain, whereas significant sustained improvement was seen at 3 and 6 months for physical functioning, and role limitations due to physical health. Emotional well-being and role functioning due to emotional problems showed peak improvement at 3 months post-operatively, whereas general health measurement peaked at 30 days. As demonstrated with disease-specific PROMs and other historic studies, this confirms the continued benefit laparoscopic cholecystectomy had on participants [26, 27].

### Validation assessments

Despite a poor overall return rate of surveys post-operatively, all returned surveys were 100% complete. This all or nothing process is more frequent with digital surveys where the ability to skip questions can be removed, or users can be prevented from progressing. Other than high response rates, the additional benefits are that missing or spurious data points do not need to be accounted for, and there are lower chances of imputation error [8]. This level of acceptability and ease of ePROM dissemination are in keeping with existing evidence which has shown similar benefits when compared to more traditional PROM dissemination methods [10, 28, 29].

Assessments to validate CSQ and SF36 for use as ePROs in our study population demonstrated strong reliability scores for CSQ across all domains, with good internal consistency scores. Comparatively, SF36 demonstrated strong reliability scores with good-to-excellent measures of internal consistency. These results were similar to those described by the original CSQ research group [21], and the Oxford PROM Group review [17], suggesting that the PROs were accurate in measuring what they set out to and that they were stable in their measurements across the different time points.

The low percentages for floor and ceiling effects in both CSQ and SF36 surveys, and the medium-to-large effect sizes, also confirmed sufficient scale variance and responsiveness suggesting suitability to continue using them as ePROs and measure the wide variability of change in HRQoL.

The moderate-to-strong cross-correlation between surveys in this study demonstrates that future platforms may be justified in offering patients a single HRQoL survey to reduce survey fatigue.

### Strengths and weaknesses

The benefit of using a digital platform meant that the PROs were easily disseminated to participants for completion at the correct time points, with an easy system in place to facilitate reminders for non-responders. The digital modality also meant that participants had access to complete HRQoL surveys in real time at any time and place, and from any digital medium which allowed Internet access. This proved especially convenient for participants who had already returned to work.

Results from completed ePROMs were also immediately available with no concerns about managing missing results, imputation errors, or researcher bias which may occur when collecting PROMs by more traditional means [8].

Although the results from this study suggest significant improvements in quality of life following laparoscopic cholecystectomy in our patient population, the study is significantly underpowered to be able to draw any reliable conclusions given the limited number of patients who completed both pre- and post-operative questionnaires. It is not possible to assume that all participants who suffered complications completed post-operative surveys and, therefore, this reporting bias could have impacted the overall results.

Despite initial problems with study set-up and participant recruitment, a large proportion of participants still engaged actively with the study process with positive interactions and acceptability demonstrating a place for digital mediums in delivering ePROMs. Although the use of ePROMs removes many of the logistic and cost issues associated with more traditional paper or telephone versions, access to ePROMs systems needs to be more inclusive and usable for both patients and clinicians [5, 30, 31]. Whilst further developmental costs may be necessary, this is fundamental to improve survey response rates and reduce recruitment bias.

### Future work and recommendations

This feasibility study has helped to provide the groundwork and real-world data necessary to plan for a powered randomised controlled trial across more than one region. Further studies should also aim to actively involve patient advisory groups in study planning to develop study protocols and help test any digital medium prior to study commencement. The inclusion of focus groups within new studies would also be beneficial in recording patient attitudes, in particular reasons for non-participation.

## Supplementary Information

Below is the link to the electronic supplementary material.

Supplementary file1 (DOCX 91 kb)
