# Supplementary material for: Utilising Electronic PROMs to Measure a Change in Health Following Elective Laparoscopic Cholecystectomy: A Feasibility Study
Source: World J Surg. 2022 May 24;46(9):2155–65. doi: 10.1007/s00268-022-06588-9 (PMC9334410; doi:10.1007/s00268-022-06588-9)
Supplement: Supplementary file 1 — Supplementary file1 (DOCX 91 kb) [file 268_2022_6588_MOESM1_ESM.docx]

**Supplementary Document**

**Supplementary Figure 1:** Correlation of ‘Physical Functioning’ between CSQ and SF36.

Preoperative, and postoperative (30-days, 3-months, 6-months) Spearman’s r correlation coefficient = 0.40, 0.41, 0.36, 0.45 (p<0.05) respectively in keeping with moderate reliability between scales.

**Supplementary Figure 2:** Correlation of ‘Systemic Functioning’ between CSQ and SF36.

Preoperative, and postoperative (30-days, 3-months, 6-months) Spearman’s r correlation coefficient = 0.68, 0.60, 0.41, 0.61 (p<0.05) respectively in keeping with moderate to strong reliability between scales.

**Supplementary Figure 3:** Correlation of ‘Emotional Functioning’ between CSQ and SF36.

Preoperative, and postoperative (30-days, 3-months, 6-months) Spearman’s r correlation coefficient = 0.72, 0.51, 0.48, 0.43 (p<0.05) respectively in keeping with moderate to strong reliability between scales.

**Supplementary Figure 4:** Correlation of ‘Social Functioning’ between CSQ and SF36.

Preoperative, and postoperative (30-days, 3-months, 6-months) Spearman’s r correlation coefficient = 0.66, 0.53, 0.59, 0.52 (p<0.05) respectively in keeping with strong reliability between scales.

**Supplementary Figure 5:** Correlation of ‘Overall health’ between CSQ and SF36.

Preoperative, and postoperative (30-days, 3-months, 6-months) Spearman’s r correlation coefficient = 0.35, 0.36, 0.35, 0.42 (p<0.05) respectively in keeping with moderate reliability between scales.

**Supplementary Table 1:** Cross correlation between CSQ and SF36 HRQoL surveys.

| **HRQoL domain** | **Spearman’s r** | **95% CI** | **p-value** |
| --- | --- | --- | --- |
| **Physical functioning** |  |  |  |
| Preoperative | **0.40^*^** | 0.28 – 0.51 | <0.01 |
| 30-days postoperative | **0.41^*^** | 0.17 – 0.60 | <0.01 |
| 3-months postoperative | **0.36^*^** | 0.10 – 0.58 | <0.01 |
| 6-months postoperative | **0.45^*^** | 0.14 – 0.68 | <0.01 |
| **Systemic functioning** |  |  |  |
| Preoperative | **0.68^**^** | 0.59 – 0.75 | <0.01 |
| 30-days postoperative | **0.60^**^** | 0.41 – 0.74 | <0.01 |
| 3-months postoperative | **0.41^*^** | 0.15 – 0.61 | <0.01 |
| 6-months postoperative | **0.61^**^** | 0.36 – 0.78 | <0.01 |
| **Emotional functioning** |  |  |  |
| Preoperative | **0.72^**^** | 0.65 – 0.78 | <0.01 |
| 30-days postoperative | **0.51^**^** | 0.29 – 0.68 | <0.01 |
| 3-months postoperative | **0.48^*^** | 0.24 – 0.67 | <0.01 |
| 6-months postoperative | **0.43^*^** | 0.12 – 0.67 | <0.01 |
| **Social functioning** |  |  |  |
| Preoperative | **0.66^**^** | 0.58 – 0.73 | <0.01 |
| 30-days postoperative | **0.53^**^** | 0.31 – 0.69 | <0.01 |
| 3-months postoperative | **0.59^**^** | 0.37 – 0.74 | <0.01 |
| 6-months postoperative | **0.52^**^** | 0.23 – 0.73 | <0.01 |
| **Overall health** |  |  |  |
| Preoperative | **0.35^*^** | 0.22 – 0.47 | <0.01 |
| 30-days postoperative | **0.36^*^** | 0.12 – 0.57 | <0.01 |
| 3-months postoperative | **0.35^*^** | 0.08 – 0.57 | 0.01 |
| 6-months postoperative | **0.42^*^** | 0.11 – 0.66 | <0.01 |

Spearman’s r: <0.30 = weak, 0.30-0.49 = moderate^*^, ≥0.5 = strong^**^ reliability. CI: Confidence Interval.
